# Androgen levels of premenopausal females are not observably associated with body composition and physical performance, but may interact with hormonal contraceptive use

**DOI:** 10.1007/s00421-025-05993-x

**Published:** 2025-09-22

**Authors:** Vera M. Salmi, Jari E. Karppinen, Terhi T. Piltonen, Heikki Kyröläinen, Juha J. Hulmi, Johanna K. Ihalainen, Ritva S. Mikkonen

**Affiliations:** 1 Faculty of Sport and Health Sciences, University of Jyväskylä, Jyväskylä, Finland; 2 Obesity Research Unit, Research Program for Clinical and Molecular Metabolism, University of Helsinki, Helsinki, Finland; 3 Department of Obstetrics and Gynaecology, Research Unit of Clinical Medicine, Medical Research Center Oulu, University of Oulu and Oulu University Hospital, Oulu, Finland; 4 Finnish Institute of High Performance Sport KIHU, Jyväskylä, Finland

**Keywords:** Androgens, Testosterone, Female physiology, Eumenorrheic, Combined oral contraceptives

## Abstract

**Purpose:**

The role of androgen levels in physiological characteristics of females is poorly understood, as previous research has mainly focused on testosterone and females not using hormonal contraceptives. Our aim was to investigate whether androgen levels are associated with body composition and physical performance in recreationally active and trained premenopausal females.

**Methods:**

The study examined two phases of the menstrual/combined oral contraceptive (COC) cycle of healthy eumenorrheic (EUM) and COC using females (age 19–35 years, *n* = 83). Total and free serum testosterone, dihydrotestosterone, androstenedione, dehydroepiandrosterone (DHEA), DHEA-sulfate, and sex hormone-binding globulin (SHBG) levels were analyzed. Linear mixed-effects models were used to examine the associations between androgen levels and fat-free mass (FFM), fat mass (FM), counter movement jump (CMJ), maximal isometric force production, and aerobic capacity (${\dot{\mathrm{V}}\mathrm{O}}$_2peak_).

**Results:**

None of the measured androgens were significantly associated with body composition or physical performance outcomes in the pooled sample. However, significant androgen–COC-use interactions indicated that the associations between DHEA and FFM (*β* = 0.23, *p* = 0.017), SHBG and FFM-FM-adjusted CMJ (*β* = 0.72, *p* = 0.041), and total testosterone and unadjusted ${\dot{\mathrm{V}}\mathrm{O}}$_2peak_ (*β* = − 0.27, *p* = 0.016) differed according to COC-use. A significant association between SHBG and CMJ (*β* = − 0.66, *p* = 0.047) and total testosterone and ${\dot{\mathrm{V}}}$O_2peak_ (*β* = 0.15, *p* = 0.044) was found only in EUM; however, adjustment for FFM eliminated this statistical significance.

**Conclusion:**

Serum androgen levels were not robustly associated with body composition or physical performance outcomes in healthy, recreationally active and trained premenopausal females. Hormonal contraceptive status may attenuate the associations between androgens and performance, driven potentially by FFM and individuals with high androgen levels.

**Supplementary Information:**

The online version contains supplementary material available at 10.1007/s00421-025-05993-x.

## Background

Androgens influence the muscular, skeletal, and cardiovascular systems, as well as reproduction in both males and females. While testosterone, the major androgen hormone, has around 10–20 times lower serum concentrations in females than in males (Bhasin et al. 2011; Braunstein et al. 2011; Diver 2008), concentrations of testosterone and other androgens are actually higher relative to concentrations estradiol, the primary estrogen secreted by the ovaries (Burger 2002; Krüger et al. 2023). Other circulating androgens in females include dihydrotestosterone (DHT), androstenedione, dehydroepiandrosterone (DHEA), and its sulfate (DHEA-S) (Abraham 1974). Only testosterone and DHT bind to the androgen receptor, to exert their effects on target tissues by activating molecular signaling. Androstenedione, DHEA, and DHEA-S are considered androgen precursors that must be converted into more potent androgens, but also to estrogens, to express their effects (Lissaman et al. 2023; Naamneh Elzenaty et al. 2022).

In males, endogenous testosterone has been positively associated with lean mass (Mouser et al. 2016; Ye et al. 2021) and aerobic capacity (Pitteloud et al. 2005). In healthy premenopausal females, however, the evidence is conflicting. In previous cross-sectional studies, basal levels of total testosterone have not been associated with lean mass (Alexander et al. 2021) or muscle quality (Pöllänen et al. 2011), while the free fraction of testosterone in serum, expressed as free androgen index (FAI), has been positively associated with lean mass (Alexander et al. 2021). Moreover, testosterone exposure has been shown to result in a leaner body composition in young physically active females (Hirschberg et al. 2020). Testosterone exposure not only drives the synthesis of proteins (Pataky et al. 2023), increases lean mass, and decreases body fat percentage, but also has an enhancing effect on aerobic performance (Hirschberg et al. 2020). At physiological levels, FAI has been positively associated with resistance training-induced muscle strength in untrained premenopausal females (Alexander et al. 2024). Similarly, basal testosterone levels have been positively associated with vertical jump performance in female athletes (Cardinale and Stone 2006).

In females, androgens are secreted from the ovaries and adrenal glands, and produced via peripheral conversion from precursors (de Jong et al. 1974; Lloyd et al. 1971; Mikhail 1970; Piltonen et al. 2002). In females, the small fraction of 0.3–2% total testosterone is circulating in the blood in its active form (free testosterone), while the rest circulates bound to albumin or sex hormone-binding globulin (SHBG) (Fiers et al. 2018; Mathor et al. 1985). Use of combined oral contraceptives (COC) affects the cyclical variation in sex hormones by suppressing serum levels of estradiol, progesterone (Mishell et al. 1972), and androgens, while increasing SHBG levels (Thorneycroft et al. 1999; Van Der Vange et al. 1990; Wiegratz et al. 2003). This anti-androgenic effect depends on the androgenicity of the progestogen in each formulation of COC (Burrows and Peters 2007). Also, natural estrogens have less estrogenic effects compared to synthetic ethinyl estradiol (EE) (Haverinen et al. 2022; Kangasniemi et al. 2022). When compared with eumenorrheic females, females using COCs appear to have slightly inferior performance (Elliott-Sale et al. 2020), however, it is currently unknown if this can be attributed to the downregulated concentrations of androgens.

Research focusing on different androgen hormones in females, rather than testosterone alone, is scarce. Furthermore, the role of endogenous androgen concentrations on body composition and physical performance in recreationally active and trained eumenorrheic females with no known gynecological problems and in females using COCs is poorly understood. Concentrations of androgens fluctuate during a normal menstrual cycle (Braunstein et al. 2011; Bui et al. 2013; Rothman et al. 2011; Salonia et al. 2008), which has generally not been considered for in previous studies. Therefore, our aim was to investigate whether endogenous serum androgen levels are associated with body composition and physical performance in healthy, recreationally active and trained premenopausal females.

## Methods

### Participants

This study uses data from two studies: “the endogenous and exogenous hormones and performance in women (MEndEx) study” and “the women’s menstrual cycle and endurance training (NaisQs) study” conducted in the University of Jyväskylä, Finland. Healthy 18–35 years old recreationally active or trained (tier 1–2) (McKay et al. 2022) females were recruited via advertisements in sport halls, gyms, and public places as well as the University of Jyväskylä’s mailing lists, website, and social media channels. When participants volunteered for the study, they completed a health questionnaire that was screened and approved by a medical doctor prior to enrollment. Inclusion criteria were as follows: a self-reported regular 26–35-day menstrual cycle or combined monophasic hormonal contraceptive use. Participants were excluded if they smoked, were breastfeeding, were unable to perform running exercises, had a chronic disease or endocrine disorder, or reported taking pharmacological agents known to affect metabolic or exercise-related responses (with the exception of combined oral contraceptives). These agents included antidepressants (such as selective serotonin reuptake inhibitors and serotonin–norepinephrine reuptake inhibitors), thyroid hormone preparations, antirheumatic drugs, and central nervous system stimulants. Further exclusion criteria were amenorrhea, clinically diagnosed polycystic ovary syndrome (PCOS) or any other disease or condition that could affect ovarian function. The study followed the principles of the Declaration of Helsinki, and the Ethical Committee of the University of Jyväskylä approved the methodology of both, MEndEx (October 22, 2018) and NaisQs (1519/13.00.04/2021) studies. Participants received detailed information about the study design, measurements, procedures, and possible risks, and all participants signed a written informed consent before the study onset.

**Fig. 1:**
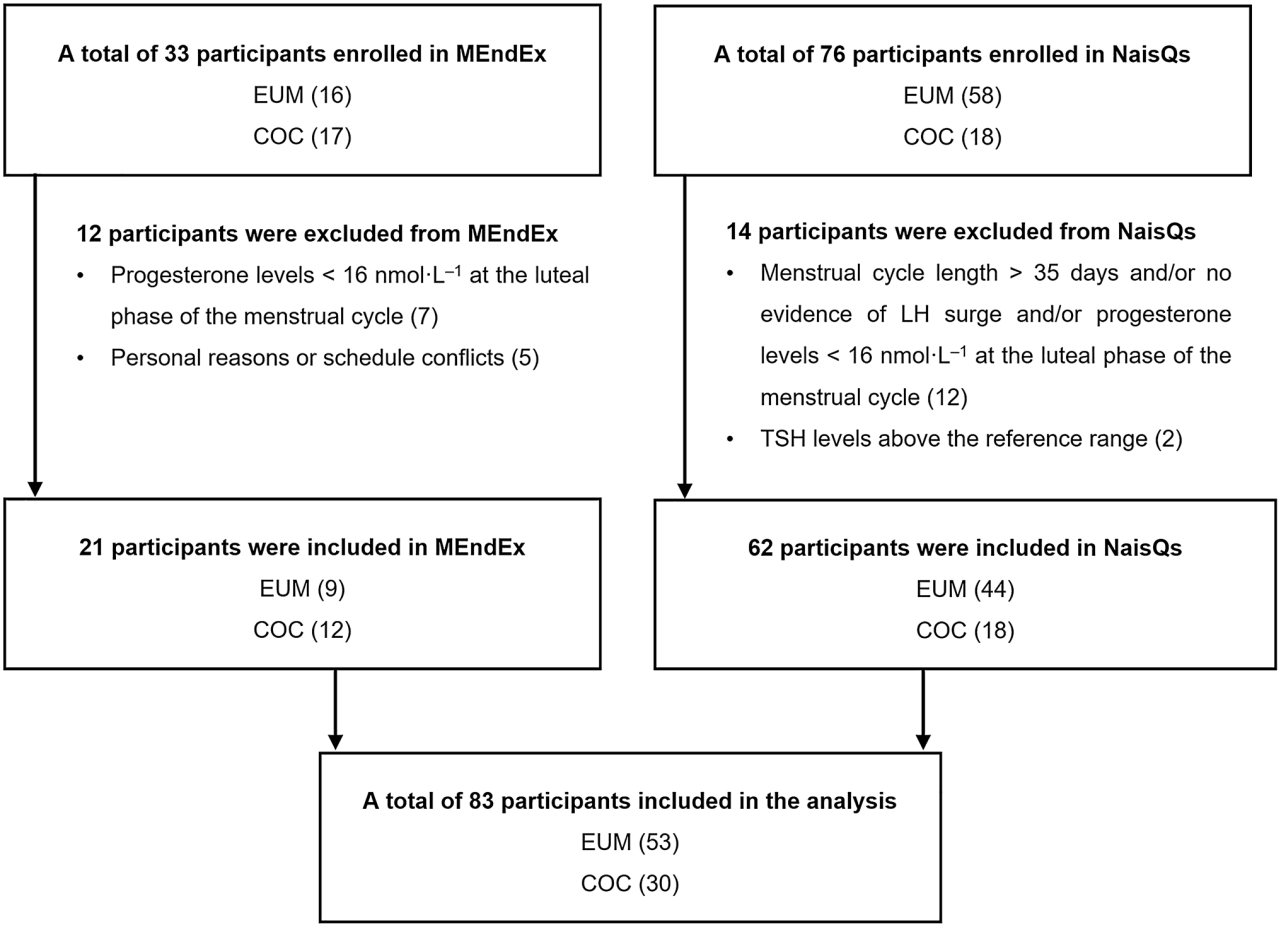
Flowchart illustrating an overview of the enrollment process of eumenorrheic females (EUM) and females using combined oral contraceptives (COC) for the MEndEx and the NaisQs study. *LH* luteinizing hormone, *TSH* thyroid stimulating hormone

A total of 109 participants were enrolled to the study. From 74 naturally menstruating females, 21 participants (28%) were excluded for not meeting the criteria for eumenorrhea (19) (Elliott-Sale et al. 2021), or having thyroid stimulating hormone levels above the reference range (2). Eumenorrhea was defined according to criteria by Elliott-Sale et al. (2021): menstrual cycle lengths ≥ 21 days and ≤ 35 days, plus evidence of the LH surge, plus correct hormonal profile, i.e., progesterone levels > 16 nmol·L^−1^ at the luteal phase of the menstrual cycle, plus no hormonal contraceptive (HC) use 3 months prior to recruitment. Two participants were included, even though they did not have a positive LH surge test, as they had serum progesterone > 16 nmol·L^−1^ at the luteal phase measurement indicating ovulation. From 35 females using COC, five participants dropped out due to personal reasons or for not adhering to the study schedule. Females using COCs had used combined monophasic oral contraceptives for at least 3 months prior to the study. The participants in the COC group used differing amounts of estrogenic (0.02–0.03 mg EE or 1.5 mg estradiol as hemihydrate) and second-, third-, or fourth-generation progestin or cyproterone acetate (0.075–3.0 mg) containing pills for 21 or 24 days (active phase) followed by 4 or 7 hormone free days (inactive phase) (Supplementary Table S1). Finally, 88 participants fulfilling the inclusion criteria were included in the analysis: 53 eumenorrheic females (EUM) and 30 females using combined oral contraceptives (COC). Figure 1 provides an overview of the enrollment process for the MEndEx and NaisQs studies.

### Study design and menstrual cycle/hormonal contraceptive phase definitions

Menstrual/hormonal contraceptive cycle tracking began on the first day of the menstrual cycle in the EUM group or the first day of the active phase, i.e., pill taking phase in the COC group. All participants filled menstrual/contraceptive diaries daily and participants in EUM were instructed to measure LH surge from the urine (described below). All participants came to laboratory measurements for one day (MEndEx) or two consecutive days (NaisQs) in two different phases of the menstrual cycle and COC pill use. The first measurement day included blood samples and body composition in fasted state. Performance tests were performed under fed conditions after fasting measurements (MEndEx) or on a second measurement day (NaisQs). In EUM, blood samples were collected in between 1 and 7 days of the follicular phase of the menstrual cycle and in the luteal phase 3–9 days (mean 7 ± 1 days) after the LH surge according to methodological recommendations by Elliott-Sale et al. (2021). In COC, the corresponding measurements were scheduled during the 1st–7th day of the inactive (i.e., pill-free or placebo) phase and the 1st–8th day of the active phase (i.e., pill taking phase).

### Blood samples

Blood samples were collected from the antecubital vein into serum tubes (9 mL Venosafe, Terumo Medical Co., Belgium or 2 × 6 mL Vacuette, Greiner Bio-One GmBH, Kremsmünster, Austria) for hormone analyses between 06.00 and 11.00 in the morning after at least 10 h of overnight fasting, abstaining 48 h from alcohol, and 24 h from strenuous exercise. Serum tubes were stored 15 min at room temperature, after which tubes were centrifuged for 10 min at 2000×*g* (Venosafe tubes) and 2245×*g* (Vacuette tubes) (Megafuge, 1.0R, Heraeus, Germany). The serum was separated and immediately frozen first at − 20 °C and later stored at − 80 °C until the final analysis. Concentrations of estradiol and progesterone were measured for validation of menstrual cycle phase. Estradiol, progesterone, total testosterone, androstenedione, DHEA-S, and SHBG were analyzed from serum using chemical luminescence techniques by Immulite®2000 XPi-analyzator (Siemens Healthcare Diagnostics, New York, USA). Free testosterone (Testosterone free ELISA, Demeditec or Testosterone free, ELISA, Biovendor), DHT [Dihydrotestosterone (DHT) ELISA], and DHEA (DHEA ELISA, Demeditec) were analyzed using enzyme-linked immunosorbent assay (ELISA) with Dynex DS2®-analyzator (Dynex Technologies, Chantilly, VA, USA). Inter-assay coefficients of variation (CV) in our laboratory were 9.7% and 8.7% for estradiol, 12.2% and 15.5% for progesterone, 6.7% for total testosterone, 12.4% for free testosterone, 14.8% for DHT, 11.2% for androstenedione, 6.9% and 4.7% for DHEA, 9.8% and 7.3% for DHEA-S, and 7.6% and 5.5% for SHBG in MEndEx and NaisQs studies, respectively. The analytical sensitivity was 55.0 pmol·L^−1^ for estradiol, 0.3 nmol·L^−1^ for progesterone, 0.5 nmol·L^−1^ for total testosterone, 0.02 nmol·L^−1^ for DHT, 1.0 nmol·L^−1^ for androstenedione, 0.08 µmol·L^−1^ for DHEA-S, and 0.02 nmol·L^−1^ for SHBG. For free testosterone, the analytical sensitivity was 0.14 pmol·L^−1^ and 0.06 pmol·L^−1^ and for DHEA 0.24 nmol·L^−1^ and 0.28 nmol·L^−1^ in MEndEx and NaisQs studies, respectively.

### LH surge test

LH surge was assessed using a urinary LH test (Dipro, LH Ovulation Strip, Aidian Oy, Finland) in the MEndEx study. In the NaisQs study, urinary concentrations of LH and estrone-3-glucuronide (metabolite of estradiol) were monitored with Clearblue dual hormone ovulation kits [Clearblue® Advanced Digital Ovulation Test, SDP Swiss Precision Diagnostics GmbH (SDP), Geneva, Switzerland]. Participants completed testing at home according to the manufacturer’s instructions and reported results to research staff to assess menstrual status and determine measurement schedules.

### Anthropometrics and body composition

Anthropometric measurements were taken in the morning after an overnight fast of at least 10 h. The height was measured with a wall-mounted stadiometer. Body composition and body mass were assessed using a multifrequency bioelectrical impedance device (Inbody 770, Biospace Co. Ltd., Seoul, Korea) with participants wearing underwear. Body mass index (BMI) was calculated as body mass (kg) divided by height squared (m^2^).

### Countermovement jump and isometric leg press

Participants were instructed to refrain from strenuous exercise for the 24 h prior to performance testing, but fasting measurements completed the day before performance tests included a peak fat oxidation test (walking on treadmill with increasing gradient) in the NaisQs study. Before performance measurements, each participant performed a warm-up session including a 3-min walk on a treadmill followed by dynamic movements (10 squats, 10 lunges, 10 side lunges, and 10 calf raises). Counter movement jump height (CMJ) (Bosco et al. 1983) was measured by flight time with photocells (Faculty of Sport and Health Sciences, University of Jyväskylä, Finland). First, participants performed 2–3 warm-up jumps followed by a minimum of three maximal effort jumps with 60-s rest between attempts until the result no longer improved. Participants were instructed to jump as high as possible while holding their hands on their hip to minimize the influence of arm swing on the performance.

Isometric force production of the leg extensors was measured with isometric horizontal bilateral leg press (Faculty of Sport and Health Sciences, University of Jyväskylä, Finland). The knee angle was 107° (Häkkinen et al. 1998), which was determined from the greater trochanter, lateral tibiofemoral joint space, and lateral malleolus. Participants were instructed to produce as much force as possible as quickly as possible for ~ 3–5-s. Participants were instructed to perform three warm-up attempts (1st 50%, 2nd 75%, and 3rd 90% of maximum force, 60-s rest between attempts), and then, a minimum of three maximal contractions were performed with 120-s rest between attempts from each participant until maximal force was obtained. Participants were verbally encouraged in every attempt. Force data were collected at a sampling frequency of 2000 Hz (Signal 4.04, CED, United Kingdom) using customized scripts (Matlab R2023b, The MathWorks Inc., Natick, MA, USA). The maximal bilateral isometric force production (*F*_max_) was determined as the difference between the zero level and the highest value of force recorded.

### Incremental treadmill running test

Peak oxygen uptake (${\dot{\mathrm{V}}}$O_2peak_) was used to assess the endurance performance level of the participants. A treadmill test was performed after strength tests using a standard incremental protocol with 3-min stages and 1 km·h^−1^ speed increasements every third minute until volitional exhaustion. Treadmill speed was 6 km·h^−1^ for the first 3-min stage of the test and incline of the treadmill remained constant at 0.6° for the entire test. The treadmill was briefly stopped for ~ 30-s between each stage for fingertip blood samples. Participants were verbally encouraged throughout the test. Oxygen consumption (${\dot{\mathrm{V}}}$O_2_) was measured breath-by-breath using a gas analyzer calibrated according to the manufacturer’s instructions before every test (Vyntus CPX, Vyaire Medical GmbH, Hoechberg, Germany). ${\dot{\mathrm{V}}}$O_2peak_ was defined as the highest rolling average 60-s ${\dot{\mathrm{V}}}$O_2_ value using K-lab 3.1.25 software (2121.10.01 Aino Health Management Oy, Helsinki).

### Statistical analysis

Data were analyzed using IBM SPSS 30.0.0.0 (SPSS Inc., Chicago, IL) and figures were prepared using GraphPad Prism 9.5.1 (GraphPad Software Inc., California, USA). Descriptive data are presented as means with standard deviations for normally distributed variables or medians with interquartile range for non-normal variables. Normality was evaluated by visual inspection of histograms and Q–Q plots.

Differences between study groups and between menstrual cycle and/or COC phases were examined with independent-samples and paired-samples Student’s *t* test for normally distributed variables and Mann–Whitney *U* test and Wilcoxon signed-rank test for non-normally distributed variables.

Associations between hormones and outcomes were investigated using linear mixed-effects models. Each model included one standardized hormone explanatory variable, standardized outcome, and a random intercept for each participant. Separate models were used to estimate the association of each hormone with outcomes due to sequential nature of conversion of DHEA and androstenedione to testosterone (Horton and Tait 1966; Zhou et al. 2015) and from androstenedione and testosterone further to DHT (Silva et al. 1987).

To increase statistical power, EUM and COC groups and different menstrual cycle or COC phases were analyzed together while adjusting for their potential confounding effects, because they might affect both the explanatory variables [hormone levels (Braunstein et al. 2011; Bui et al. 2013; Endrikat et al. 2002; Goebelsmann et al. 1974; Kangasniemi et al. 2022; Rothman et al. 2011; Salonia et al. 2008] and outcomes [body composition (Casazza et al. 2002) and physical performance (Elliott-Sale et al. 2020; McNulty et al. 2020)]. The inactive pill phase was aligned with the follicular phase due to similar hormonal environments (low concentrations of estradiol and progesterone) between phases. The active pill phase was aligned with the luteal phase, while acknowledging that the hormonal environments differ between the two groups at these time points (Elliott-Sale et al. 2021). To test whether the use of COC moderated the hormone–outcome associations, hormone–COC-use interaction was included in separate models.

To adjust for body size and composition, which could confound or mediate the hormone–outcome relationships, following covariate structure was applied. Height was included as a covariate when fat-free mass (FFM) or fat mass (FM) was the outcome. FM was also analyzed with FFM as a covariate to isolate associations with relative adiposity. Performance-outcome models were run first unadjusted and then adjusted for body composition. Specifically, CMJ models included both FFM and FM as a covariates, whereas *F*_max_ and absolute ${\dot{\mathrm{V}}}$O_2peak_ models included FFM only. All covariates were standardized before analysis.

The model assumptions were verified before accepting the results and statistical significance was set at *p* < 0.05. Because the hormone variables included some extreme observations that could skew the results, sensitivity analyses were performed after excluding those values that exceeded both 1.5 × interquartile range (Walfish 2006; Yang et al. 2019) and an absolute *Z*-score > 3. However, no physiological or measurement-related reasons for these observations were found. Excluded values of total testosterone (Al Kindi et al. 2012; Bui et al. 2015; Georgopoulos et al. 2014; Kumar et al. 2005), free testosterone (Bui et al. 2015; Huang et al. 2022; Kumar et al. 2005; Søeborg et al. 2014; Van Der Vange et al. 1990), androstenedione (Georgopoulos et al. 2014), DHEA-S (Khan et al. 2021; Kumar et al. 2005), and SHBG (Wiegratz et al. 2003) were considered physiologically plausible, although most of the previously reported corresponding values have been reported in participants with PCOS. Hormone values excluded for sensitivity analysis (one total testosterone, androstenedione and DHEA-S value, three DHT, DHEA, and SHBG values, and four free testosterone values) are presented in Supplementary Table S2.

## Results

### Participant characteristics and hormonal differences between phases

The EUM and COC groups were largely similar on all baseline characteristics except age and BMI as participants in the COC group were younger and had lower BMI than those in the EUM group (Table 1). Within the EUM group, estradiol, progesterone, and SHBG concentrations were higher in the luteal phase, whereas free testosterone and DHT concentrations were higher in the follicular phase (Table 2). In contrast, hormone concentrations among COC users were generally stable across the phases, except that SHBG concentrations were higher in the inactive phase (Table 2). No statistically significant phase-related differences in body composition or physical performance outcomes were observed in either group (Table 2).

**Table 1:** Participant characteristics in eumenorrheic females (EUM) at follicular phase and in females using combined oral contraceptives (COC) at inactive phase including group comparisons

Group	EUM (*n* = 46–53)	COC (*n* = 28–29)	p*
Age (years)	29.3 ± 4.1	24.9 ± 4.0	< 0.001
Height (m)	1.67 ± 0.06	1.69 ± 0.05	0.099
Weight (kg)	68.4 ± 10.3	66.5 ± 7.1	0.398
BMI (kg·m^−2^)	24.7 ± 3.8	23.3 ± 2.3	0.046
Body fat percentage (%)	27.0 ± 8.2	25.1 ± 6.8	0.296
Length of MC or COC phases (days)	27 ± 2	21–24 active + 4–7 inactive	
Day of ovulation	14 ± 2		

Values presented as mean ± standard deviation

*BMI* body mass index, *MC* menstrual cycle

*The *p* values are from independent-samples Student’s *t *test

**Table 2:** Serum hormones, body composition, and physical performance in eumenorrheic females (EUM) across menstrual cycle phases and in females using combined oral contraceptives (COC) across pill phases

	EUM		COC			
	Follicular phase	Luteal phase	Inactive phase	Active phase	Follicular vs. inactive phase	Luteal vs. active phase
Hormones	(*n* = 45–46^a^)	(*n* = 52–53^a^)	(*n* = 28^a^)	(*n* = 28–30^a^)	*p*^#^	*p*^#^
Estradiol (pmol·L^–1^)	124.5 (85.9–184.3)	**510**.**0** (**413**.**0**–**721**.**5**)***	100.4 (42.6–180.5)	98.7 (58.1–177.8)	0.090	<** 0**.**001**
Progesterone (nmol·L^–1^)	1.3 (0.9–1.8)	**25**.**8** (**21**.**5**–**34**.**0**)***	1.0 (0.6–1.4)	1.0 (0.8–1.2)	0.063	<** 0**.**001**
Total testosterone (nmol·L^–1^)	0.7 (0.5–1.0)	0.8 (0.4–1.2)	0.8 (0.4–1.6)	1.0 (0.6–1.1)	0.238	0.296
Free testosterone (pmol·L^–1^)	8.4 (5.0–11.6)	**8**.**0 **(**4**.**5**–**11**.**1**)*	5.8 (3.4–8.0)	5.6 (4.2–9.3)	**0.048**	0.152
DHT (nmol·L^–1^)	2.3 (1.7–2.6)	**2**.**1** (**1**.**6**–**2**.**6**)*	1.9 ± 1.2	2.0 ± 1.1	0.159	0.313
Androstenedione (nmol·L^–1^)	8.5 ± 4.1	9.2 ± 3.9	9.7 ± 5.1	11.1 ± 5.1	0.542	0.175
DHEA (nmol·L^–1^)	44.3 (26.5–69.4)	41.5 (28.4–73.4)	39.5 (29.2–57.6)	38.2 (27.0–69.6)	0.789	0.809
DHEA-S (µmol·L^–1^)	5.1 ± 2.3	5.2 ± 2.2	4.8 ± 2.2	4.8 ± 2.3	0.713	0.396
SHBG (nmol·L^–1^)	59.2 (41.3–69.8)	**62**.**0** (**44**.**1**–**70**.**9**)**	210.5 (170.8–272.3)	**156**.**0** (**120**.**3**–**208**.**8**)***	** < 0.001**	<** 0**.**001**

Body composition	(*n* = 46)	(*n* = 53)	(*n* = 28)	(*n* = 29)	*p*^#^	*p*^#^
Fat-free mass (kg)	49.4 ± 5.4	49.1 ± 5.1	49.6 ± 5.4	49.4 ± 5.1	0.843	0.833
Fat mass (kg)	19.0 ± 8.1	19.2 ± 8.3	16.9 ± 5.7	16.7 ± 5.6	0.226	0.153

Physical performance	(*n* = 42–43)	(*n* = 48–49)	(*n* = 25–26)	(*n* = 27–28)	*p*^#^	*p*^#^
*F*_max_ (N)	2640 ± 664	2692 ± 614	2641 ± 762	2653 ± 736	0.994	0.805
*F*_max_ (N·kg^−1^ weight)	38.8 ± 8.0	39.1 ± 7.8	39.5 ± 9.8	40.2 ± 9.4	0.743	0.571
CMJ (cm)	24.2 ± 5.8	25.1 ± 6.4	26.7 ± 4.7	26.3 ± 4.5	0.074	0.358
${\dot{\mathrm{V}}}$O_2peak_ (L·min^−1^)	2.6 ± 0.4	2.7 ± 0.4	2.8 ± 0.3	2.8 ± 0.3	0.153	0.225
${\dot{\mathrm{V}}}$O_2peak_ (mL·kg^−1^·min^−1^)	38.9 ± 6.0	38.7 ± 6.1	40.8 ± 5.1	41.7 ± 5.6	0.191	**0**.**036**

Values presented as mean ± standard deviation or median and interquartile range (25th–75th percentile)

*CMJ* counter movement jump height, *DHT* dihydrotestosterone, *DHEA* dehydroepiandrosterone, *DHEA-S* dehydroepiandrosterone sulfate, *F*_*max*_ maximal bilateral isometric force production, *SHBG* sex hormone-binding globulin, $\dot{V}$*O*_*2peak*_ peak oxygen uptake

*p < 0.05, **p < 0.01, ***p < 0.001 for differences between the follicular and luteal phases of the menstrual cycle and between the inactive and active phases of the combined oral contraceptive use from paired-samples Student’s *t *test (mean ± standard deviation) and Wilcoxon signed-rank test (median and interquartile range)

^#^The *p *values are from the independent-samples Student’s *t *test (mean ± standard deviation) and Mann–Whitney *U* test (median and interquartile range)

Significant associations are in bold

^a^For androstenedione and DHT, *n* = 37 at follicular phase, *n* = 44 at luteal phase, and *n* = 18 at active and inactive phases

### Associations of androgen concentrations with body composition

Across all observations, i.e., both groups at both menstrual cycle/hormonal contraceptive phase time points, none of the androgens or SHBG were associated with FFM or FM (Table 3). Hormone–COC-use interaction terms showed that these non-significant associations were consistent between the EUM and COC groups, suggesting that combining the two groups was acceptable. However, the DHEA–COC-use interaction was significant for FFM (*β* = 0.23, *p* = 0.017), indicating that the association between DHEA and FMM might differ between the groups (Table 4). Within-group estimates indicated non-significant association in the EUM group (*β* = − 0.07, *p* = 0.326) and positive association in the COC group (*β* = 0.16, *p* = 0.015). Excluding extreme hormone values in the sensitivity analyses did not materially change the results.

**Table 3:** Associations of androgen and SHBG concentrations with fat-free mass and fat mass

	Fat-free mass		Fat mass	
	Std. estimate (95% CI)	*p*	Std. estimate (95% CI)	*p*
Total testosterone	− 0.017 (− 0.072, 0.037)	0.529	− 0.002 (− 0.037, 0.033)	0.909
Free testosterone	0.004 (− 0.079, 0.086)	0.931	− 0.021 (− 0.079, 0.036)	0.460
DHT	0.029 (− 0.086, 0.143)	0.621	− 0.025 (− 0.114, 0.063)	0.571
Androstenedione	0.003 (− 0.063, 0.070)	0.917	− 0.036 (− 0.078, 0.007)	0.097
DHEA	0.051 (− 0.045, 0.148)	0.294	− 0.062 (− 0.130, 0.006)	0.074
DHEA-S	0.003 (− 0.073, 0.078)	0.942	− 0.044 (− 0.094, 0.007)	0.090
SHBG	− 0.053 (− 0.149, 0.042)	0.268	0.041 (− 0.020, 0.103)	0.182

*DHT* dihydrotestosterone, *DHEA* dehydroepiandrosterone, *DHEA-S* dehydroepiandrosterone sulfate, *SHBG* sex hormone-binding globulin

Estimates, confidence intervals (CI), and *p* values are from linear mixed-effects models with random intercepts for participants

**Table 4:** Differences in hormone associations with fat-free mass and fat mass in combined oral contraceptive using females compared with eumenorrheic females

	Fat-free mass		Fat mass	
	Std. estimate (95% CI)	*p*	Std. estimate (95% CI)	*p*
Total testosterone	− 0.031 (− 0.142, 0.081)	0.587	0.034 (− 0.038, 0.105)	0.351
Free testosterone	0.061 (− 0.151, 0.273)	0.568	− 0.027 (− 0.174, 0.120)	0.719
DHT	− 0.038 (− 0.308, 0.231)	0.778	0.102 (− 0.097, 0.300)	0.311
Androstenedione	0.023 (− 0.122, 0.168)	0.754	− 0.021 (− 0.115, 0.073)	0.659
DHEA	**0**.**231** (**0**.**043**, **0**.**420**)	**0**.**017**	− 0.008 (− 0.147, 0.130)	0.905
DHEA-S	− 0.056 (− 0.242, 0.130)	0.553	0.074 (− 0.056, 0.204)	0.261
SHBG	0.155 (− 0.312, 0.623)	0.512	0.106 (− 0.233, 0.445)	0.537

*DHT* dihydrotestosterone, *DHEA* dehydroepiandrosterone, *DHEA-S* dehydroepiandrosterone sulfate, *SHBG* sex hormone-binding globulin

Estimates (hormone and combined oral contraceptive use interactions), confidence intervals (CI), and *p* values are from linear mixed-effects models with random intercepts for participants

Significant associations are in bold

### Associations of androgen concentrations with physical performance

Across the pooled data set, none of the androgens or SHBG were associated with physical performance outcomes (Table 5). In a sensitivity analysis excluding the extreme hormone values, a significant negative association between DHEA-S and ${\dot{\mathrm{V}}}$O_2peak_ was observed (*β* = − 0.15, *p* = 0.038), but not after FFM adjustment (*β* = − 0.01, *p* = 0.878). For performance-outcome models adjusted for body composition, see Supplementary Table S3 and S4.

**Table 5:** Associations of androgen and SHBG concentrations with physical performance

	Counter movement jump height (CMJ)		Maximal bilateral isometric force production (*F*_max_)		Aerobic capacity (${\dot{\mathrm{V}}}$O_2peak_)	
	Std. estimate (95% CI)	*p *value	Std. estimate (95% CI)	*p *value	Std. estimate (95% CI)	*p *value
Total testosterone	0.029 (− 0.079, 0.136)	0.599	− 0.026 (− 0.129, 0.078)	0.623	0.027 (− 0.084, 0.139)	0.627
Free testosterone	− 0.070 (− 0.212, 0.072)	0.332	0.012 (− 0.126, 0.151)	0.859	0.053 (− 0.086, 0.191)	0.453
DHT	− 0.040 (− 0.247, 0.166)	0.699	0.057 (− 0.123, 0.236)	0.534	− 0.020 (− 0.173, 0.132)	0.793
Androstenedione	0.012 (− 0.118, 0.141)	0.859	0.007 (− 0.098, 0.112)	0.894	− 0.041 (− 0.143, 0.061)	0.426
DHEA	0.091 (− 0.070, 0.253)	0.266	0.046 (− 0.111, 0.204)	0.562	− 0.153 (− 0.311, 0.005)	0.058
DHEA-S	0.088 (− 0.049, 0.225)	0.207	− 0.013 (− 0.146, 0.120)	0.850	− 0.106 (− 0.240, 0.029)	0.123
SHBG	0.049 (− 0.139, 0.237)	0.605	− 0.161 (− 0.341, 0.020)	0.080	0.065 (− 0.124, 0.253)	0.498

*DHT* dihydrotestosterone, *DHEA* dehydroepiandrosterone, *DHEA-S* dehydroepiandrosterone sulfate, *SHBG* sex hormone-binding globulin

Estimates , confidence intervals (CI), and *p *values are from linear mixed-effects models with random intercepts for participants

**Fig. 2:**
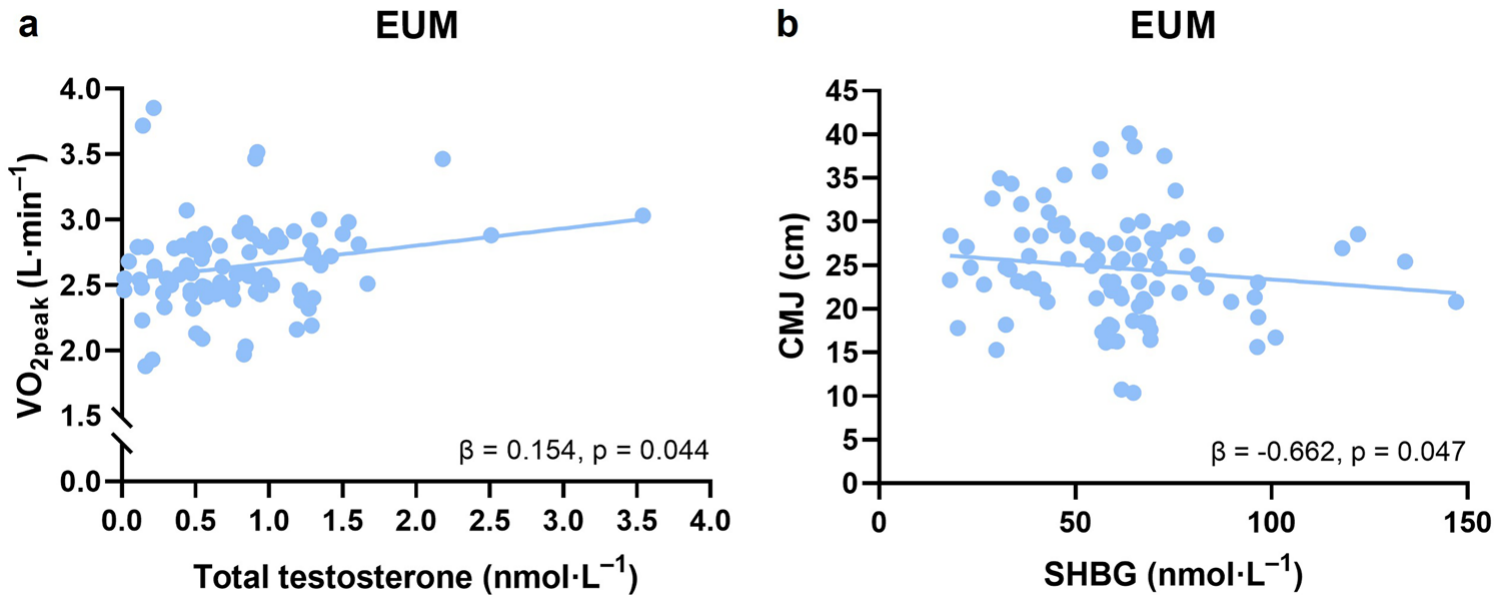
Associations between total testosterone and aerobic capacity (${\dot{\mathrm{V}}}$O_2peak_) (**a**) and between sex hormone-binding globulin (SHBG) and counter movement jump (CMJ) (**b**) in eumenorrheic females (EUM)

Adding hormone–COC-use interaction terms identified two significant interactions in the CMJ model adjusted for both FFM and FM: DHT–COC-use (*β* = 0.45, *p* = 0.030) and SHBG–COC-use (*β* = 0.72, *p* = 0.041) (Supplementary Table S4). The within-group estimates showed that DHT was not significantly associated with CMJ in either group (*β* = − 0.12, *p* = 0.226 for EUM and *β* = 0.33, *p* = 0.065 for COC). In contrast, SHBG was negatively associated with CMJ in the EUM group (*β* = − 0.66, *p* = 0.047, Fig. 2b) but not in the COC group (*β* = 0.06, *p* = 0.538). Sensitivity analyses did not materially alter these associations.

For absolute ${\dot{\mathrm{V}}}$O_2peak_, the total testosterone–COC-use interaction was significant (*β* = − 0.27, *p* = 0.016) (Table 6). The within-group association was positive in the EUM group (*β* = 0.15, *p* = 0.044, Fig. 2a) but non-significant in the COC group (*β* = − 0.12, *p* = 0.152). This interaction persisted in the sensitivity analysis (*β* = − 0.26, *p* = 0.031), although the association in the EUM group was no longer significant (*β* = 0.14, *p* = 0.097). Adjusting for FFM eliminated the significant interaction effect (*β* = − 0.16, *p* = 0.155 for primary analysis, and *β* = − 0.14, *p* = 0.235 for sensitivity analysis) and the within-group association in the EUM group (*β* = 0.10, *p* = 0.150 for primary analysis, and *β* = 0.09, *p* = 0.278 for sensitivity analysis) indicating that differences in FFM potentially accounted for the group disparity.

**Table 6:** Differences in hormone associations with physical performance in combined oral contraceptive using females compared with eumenorrheic females

	Counter movement jump height (CMJ)		Maximal bilateral isometric force production (*F*_max_)		Aerobic capacity (${\dot{\mathrm{V}}}$O_2peak_)	
	Std. estimate (95% CI)	*p *value	Std. estimate (95% CI)	*p *value	Std. estimate (95% CI)	*p *value
Total testosterone	− 0.098 (− 0.316, 0.121)	0.377	− 0.114 (− 0.326, 0.098)	0.287	− **0**.**271** (− **0**.**492**, − **0**.**051**)	**0**.**016**
Free testosterone	0.187 (− 0.180, 0.553)	0.315	− 0.134 (− 0.491, 0.223)	0.458	− 0.099 (− 0.457, 0.259)	0.587
DHT	0.237 (− 0.249, 0.723)	0.336	− 0.124 (− 0.539, 0.292)	0.555	0.080 (− 0.285, 0.444)	0.665
Androstenedione	− 0.081 (− 0.362, 0.201)	0.570	0.072 (− 0.157, 0.302)	0.530	− 0.113 (− 0.332, 0.107)	0.310
DHEA	− 0.045 (− 0.371, 0.281)	0.785	0.121 (− 0.196, 0.437)	0.453	0.057 (− 0.263, 0.378)	0.723
DHEA-S	− 0.196 (− 0.518, 0.126)	0.231	0.080 (− 0.228, 0.388)	0.608	− 0.184 (− 0.496, 0.128)	0.246
SHBG	0.359 (− 0.426, 1.143)	0.367	− 0.020 (− 0.778, 0.738)	0.958	0.270 (− 0.507, 1.047)	0.493

*DHT* dihydrotestosterone, *DHEA* dehydroepiandrosterone, *DHEA-S* dehydroepiandrosterone sulfate, *SHBG* sex hormone-binding globulin

Estimates (hormone and combined oral contraceptive use interactions), confidence intervals (CI), and *p *values are from linear mixed-effects models with random intercepts for participants

Significant associations are in bold

## Discussion

The aim of this study was to investigate whether endogenous androgen levels are associated with body composition and physical performance in healthy, recreationally active and trained premenopausal females and whether the use of COC moderates these associations. The main finding was the absence of credible associations between androgens and body composition or physical performance outcomes. However, COC-use appeared to modify the associations between DHEA and FFM, total testosterone and ${\dot{\mathrm{V}}}$O_2peak_, and SHBG and CMJ.

### Associations of hormone concentrations with body composition and physical performance

A positive association between higher DHEA concentrations and greater FFM was only found in the COC group. Previous studies exploring the associations between endogenous androgen levels and body composition in relation to the use of COCs are sparse and mainly performed in athletes. Eklund et al. (2017) found that DHEA was positively correlated with lean mass, CMJ, and squat jump in female athletes, suggesting that endogenous androgens are associated with a more muscular body composition and enhanced performance. Participants included both, athletes not using HC and athletes using HC, although the type of HC was not described in detail (the majority of participants used an HC type that inhibits ovulation). Previous research has shown that females using androgenic COCs (levonorgestrel) have greater increase in FFM and muscle strength in response to strength training program compared to females using anti-androgenic COCs (Ruzić et al. 2003). While Eklund et al. (2017) found a similar significant positive correlation between DHEA and lean mass variables among the subgroup of athletes not using HC, the specific correlation in HC using athletes was not reported. However, they found no statistically observable interactions between concentrations of DHEA and HC use (Eklund et al. 2017).

Compared to eumenorrheic females, COC users may, on average, have slightly inferior strength and endurance exercise performance (Elliott-Sale et al. 2020). Some of the proposed mechanism by which COCs may influence performance outcomes include effects on body composition (Casazza et al. 2002) and/or downregulated levels of endogenous hormones (Elliott-Sale et al. 2020). Findings on body composition have been inconsistent, as some studies have shown that COCs might increase body weight and fat mass possibly impacting performance outcomes (Casazza et al. 2002), while some of the studies have shown no change in body composition (Grandi et al. 2014; Mayeda et al. 2014; Procter-Gray et al. 2008; Reubinoff et al. 1995) or the effects of COCs on body composition have been seen without concurrent impact on physical performance (Rickenlund et al. 2004). For long term, COC-use has not been found to be a predictor for weight increase (Lindh et al. 2011). Interestingly, when comparing regularly menstruating female endurance athletes with athletes with menstrual disturbances (amenorrhea or oligomenorrhea), an increase in body weight and body fat mass was seen only in athletes with menstrual disturbances treated with COC (Rickenlund et al. 2004, 2010). These changes were associated with decline in free testosterone and androstenedione concentrations (Rickenlund et al. 2004).

The association between DHT levels and CMJ, adjusted for FFM and FM, appeared to differ depending on COC-use. Although DHT has been found to positively correlate to CMJ and squat jump in female athletes (Eklund et al. 2017), we did not find a significant association in either of the groups. Instead, a significant negative association between SHBG levels and CMJ was found only in eumenorrheic females. Concentrations of SHBG being negatively associated with CMJ only in EUM could be explained by the binding of testosterone to SHBG leading to lower circulating free hormone fraction (Anderson 1974), as higher total and free testosterone levels have been associated with enhanced jump performance (Cardinale and Stone 2006) and power performance (Bermon and Garnier 2017). The use of COC might have affected this association, as concentrations of SHBG are shown to increase by about 1.5–1.6-fold during the 21-day active phase induced by the action of EE (Endrikat et al. 2002). Moreover, different estrogen preparations affect SHBG (Wiegratz et al. 2003) and total testosterone levels (Haverinen et al. 2022) differently. Although participants had no known gynecological problems, participants with higher concentrations of androgens could include undiagnosed individuals, since PCOS is associated with enhanced secretion of androgens and their precursors (Goodarzi et al. 2015; Kumar et al. 2005). Indeed, in females with PCOS androgens have been associated with enhanced explosive performance (Kogure et al. 2015).

The positive association between concentrations of total testosterone and ${\dot{\mathrm{V}}}$O_2peak_ in EUM seemed to be biased and driven by one female having higher total testosterone level and greater ${\dot{\mathrm{V}}}$O_2peak_, since excluding this observation led to a non-significant association. Pataky et al. (2023) found a positive association between plasma testosterone and VO_2max_ which was driven by greater VO_2max_ in a few young females with higher concentrations of plasma testosterone. Some studies have not found a relationship between androgens and aerobic capacity in females (Keller et al. 2011). The association between total testosterone and ${\dot{\mathrm{V}}}$O_2peak_ in EUM was no longer significant when adjusted with FFM, which may indicate that FFM is a mediator of the association. Our results suggest that the negative association between DHEA-S and ${\dot{\mathrm{V}}}$O_2peak_, which became significant only after excluding a statistical outlier with a high DHEA-S concentration, may be mediated by FFM, as the association was no longer significant after adjusting for FFM. Although high testosterone levels are associated with greater ${\dot{\mathrm{V}}}$O_2peak_, other factors likely play a greater role for aerobic capacity.

Previous research has shown that COCs decrease concentrations of total and free testosterone (Haverinen et al. 2022; Wiegratz et al. 2003; Zimmerman et al. 2014) leading to lower levels compared to naturally menstruating females, which has been observed also by us (unpublished data). This effect may explain the observed difference in the association between total testosterone concentrations and ${\dot{\mathrm{V}}}$O_2peak_ between eumenorrheic and COC using females. Moreover, this difference between groups might be explained with FFM, as it was no longer significant after FFM adjustment. Testosterone exposure has been shown to increase lean mass and enhance aerobic performance (Hirschberg et al. 2020). Another mechanism behind the androgen concentrations and enhanced endurance performance could be related to hemoglobin levels, which affect aerobic capacity and ${\dot{\mathrm{V}}}$O_2max_ by increasing the transport of oxygen to muscles (Ekblom et al. 1976). Higher hemoglobin levels are also associated with iron metabolism, stimulation of erythropoietin production, and suppression of hepcidin, all processes that androgens are known to influence (Bachman et al. 2014; Warren and Grossmann 2022).

### Strengths and limitations

The strength of this research is including a broad set of different androgen hormones and their precursors, as previous research on females has mainly focused on testosterone. Although menstrual cycle or COC phase does not appear to impact body composition measured by bioelectrical impedance analysis (Rael et al. 2020; Thompson et al. 2021), all models were adjusted for menstrual/hormonal contraceptive cycle phase, as androgens and performance variables might be influenced by different phases of the cycle. Previous research, including analyses from our laboratory (unpublished data), shows that concentrations of total testosterone fluctuate during the menstrual cycle, peaking at mid-cycle (Braunstein et al. 2011; Bui et al. 2013; Goebelsmann et al. 1974; Rothman et al. 2011; Salonia et al. 2008). Moreover, exercise performance might be trivially reduced during the early follicular phase of the menstrual cycle, compared to other phases in eumenorrheic females (McNulty et al. 2020), while it seems to be consistent across the oral contraceptive phases (Elliott-Sale et al. 2020). COCs suppress endogenous production of estradiol, progesterone, and androgens (Haverinen et al. 2022; Kangasniemi et al. 2022; Zimmerman et al. 2014) and might be a potential confounding factor in the associations between hormone levels and body composition and performance outcomes. For this reason, all models were adjusted for the use of hormonal contraceptives.

It is worth considering that from naturally menstruating females who were enrolled to the study, over one fourth (26%) were excluded retrospectively prior to analysis due to not meeting the criteria for eumenorrhea (menstrual cycle lengths ≥ 21 days and ≤ 35 days, plus evidence of the LH surge, plus correct hormonal profile, i.e., progesterone levels > 16 nmol·L^−1^ at the luteal phase of the menstrual cycle) based on Elliott-Sale et al. (2021). Even though menstrual and hormonal contraceptive phase was standardized, the timing of blood sample collection may have influenced the observed hormone concentrations in the EUM. In EUM, blood samples were collected during the follicular phase of the menstrual cycle 1–7 days following the onset of bleeding and in the luteal phase of the menstrual cycle 3–9 days (mean 7 ± 1 days) after the LH surge. Due to cyclic nature of fluctuations in androgens during menstrual cycle, especially toward mid-cycle and expected ovulation, the variation of the timing in follicular phase measurement might have slightly affected the androgen levels. The used immunoassay method can be considered as limitation given higher sensitivity with mass spectrometry for investigating testosterone concentrations in females (Kanakis et al. 2019; Taieb et al. 2003). It should also be noted that bioelectrical impedance analysis (BIA), which was used to assess body composition, can overestimate fat-free mass while underestimating fat mass when compared with dual-energy X-ray absorptiometry (DXA), which is usually considered as the gold standard method for measuring body composition (Achamrah et al. 2018). However, fat-free mass and fat mass measured by BIA correlate strongly with corresponding DXA measures (Achamrah et al. 2018; Potter et al. 2022), making these the most accurate BIA metrics. Finally, dose and type of oral contraceptive were not controlled for in this study; dose and androgenicity of COCs varied between participants, potentially affecting the associations between androgen levels and performance outcomes as androgenicity affects the free androgen fraction (Bergink et al. 1981) and has been suggested to impact performance (Ruzić et al. 2003).

### Future perspectives

Future research investigating the associations between body composition, and performance outcomes across menstrual cycle and COC phases is warranted to corroborate these findings. Potential confounders, such as physical activity and diet, which may affect hormone concentrations (Ennour-Idrissi et al. 2015; Hulmi et al. 2017; Loucks et al. 1998; Loucks and Thuma 2003), body composition (Dupuit et al. 2020; Gao et al. 2016; Hays et al. 2025; Mouad et al. 2025; Nolte et al. 2025; Sahni et al. 2015; Xie et al. 2024), and physical performance (Grgic et al. 2018) should be considered in future research. Investigating the dose and androgenicity of COCs could provide more consistent evidence regarding the effects of different COC preparations. Future studies could consider investigating relationships between androgen levels and fat-free mass and fat mass distribution. Although the accumulation of visceral adipose tissue in females appears to be associated with increased androgenicity, current knowledge on the relationship between androgens and fat-free mass/fat mass distribution is still inconsistent (Blouin et al. 2008; Leenen et al. 1994; Tchernof et al. 2018).

## Conclusions

None of the androgens were significantly associated with body composition or physical performance outcomes in recreationally active and trained eumenorrheic and combined oral contraceptive using females. However, the results indicate that hormonal contraceptive use status might moderate associations between androgens and body composition and physical performance. Androgen levels might exert physiological effects on physical performance through the effects on body composition, rather than independently, as associations observed in eumenorrheic females and females using combined oral contraceptives seemed to be linked to fat-free mass.

## Supplementary Information

Below is the link to the electronic supplementary material.

Supplementary file1 (DOCX 26 KB)

## Data Availability

The data presented in this study are available from the corresponding author on reasonable request.

## Abbreviations

BMI: Body mass index
COC: Combined oral contraceptive
CI: Confidence interval
CMJ: Counter movement jump height
CV: Coefficient of variation
DHEA: Dehydroepiandrosterone
DHEA-S: Dehydroepiandrosterone sulfate
DHT: Dihydrotestosterone
EUM: Eumenorrheic females
*F*_max_: Maximal bilateral isometric force production
FFM: Fat-free mass
FM: Fat mass
SD: Standard deviation
SHBG: Sex hormone-binding globulin
$${\dot{\mathrm{V}}\mathrm{O}}$$_2_: Oxygen consumption
${\dot{\mathrm{V}}\mathrm{O}}$_2peak_: Peak oxygen uptake
*β*: Standardized estimate
