# Correction: Androgen levels of premenopausal females are not observably associated with body composition and physical performance, but may interact with hormonal contraceptive use

**DOI:** 10.1007/s00421-025-06043-2

**Published:** 2026-03-31

**Authors:** Vera M. Salmi, Jari E. Karppinen, Terhi T. Piltonen, Heikki Kyröläinen, Juha J. Hulmi, Johanna K. Ihalainen, Ritva S. Mikkonen

**Affiliations:** 1 Faculty of Sport and Health Sciences, University of Jyväskylä, Jyväskylä, Finland; 2 Obesity Research Unit, Research Program for Clinical and Molecular Metabolism, University of Helsinki, Helsinki, Finland; 3 Department of Obstetrics and Gynaecology, Research Unit of Clinical Medicine, Medical Research Center Oulu, University of Oulu and Oulu University Hospital, Oulu, Finland; 4 Finnish Institute of High Performance Sport KIHU, Jyväskylä, Finland

**Correction: European Journal of Applied Physiology**

10.1007/s00421-025-05993-x

In the original version of this article, in the abstract, the second sentence of the methods section read: “Total and free serum testosterone, dehydroepiandrosterone, androstenedione, dehydroepiandrosterone (DHEA), DHEA-sulfate, and sex hormone-binding globulin (SHBG) levels were analyzed”. It should have read: “Total and free serum testosterone, dihydrotestosterone, androstenedione, dehydroepiandrosterone (DHEA), DHEA-sulfate, and sex hormone-binding globulin (SHBG) levels were analyzed”. In addition, the middle dot (·) was missing in units such as “nmol·L^−1^” and “pmol·L^−1^”.

In the discussion section, the citation “Haverinen et al. 2022, 2022” incorrectly displayed the year twice. The citation should read “Haverinen et al. 2022”. In the discussion section, hyperlinks were missing for the references “Haverinen et al. 2022; Wiegratz et al. 2003”. The following reference was inadvertently omitted from the reference list: “Abraham GE (1974). Ovarian and adrenal contribution to peripheral androgens during the menstrual cycle. J Clin Endocrinol Metab 39(2):340–346. 10.1210/jcem-39-2-340.

The original article has been corrected.

