# Supplementary material for: Androgen levels of premenopausal females are not observably associated with body composition and physical performance, but may interact with hormonal contraceptive use
Source: Eur J Appl Physiol. 2025 Sep 22;126(4):1937–51. doi: 10.1007/s00421-025-05993-x (PMC13171943; doi:10.1007/s00421-025-05993-x)
Supplement: Supplementary file 1 — Supplementary file1 (DOCX 26 KB) [file 421_2025_5993_MOESM1_ESM.docx]

Androgen Levels of Premenopausal Females Are Not Observably Associated with Body Composition and Physical Performance, but May Interact with Hormonal Contraceptive Use

European Journal of Applied Physiology

Vera M. Salmi^1^*, Jari E. Karppinen^2^, Terhi T. Piltonen^3^, Heikki Kyröläinen^1^, Juha J. Hulmi^1^, Johanna K. Ihalainen^1,4^, T., Ritva S. Mikkonen^1^

1. Faculty of Sport and Health Sciences, University of Jyväskylä, Jyväskylä, Finland
2. Obesity Research Unit, Research Program for Clinical and Molecular Metabolism, University of Helsinki, Helsinki, Finland
3. Department of Obstetrics and Gynaecology, Research Unit of Clinical Medicine, Medical Research Center Oulu, University of Oulu and Oulu University Hospital, Oulu, Finland
4. Finnish Institute of High Performance Sport KIHU, Jyväskylä, Finland

*Corresponding author: Vera M. Salmi, vera.m.salmi@jyu.fi

**Supplementary Table S1 Hormone components and brand names of combined oral contraceptives**

|  | **Pills included (*n*)** | **Content (mg/mg)** | **Brand names (active phase + inactive phase)** |
| --- | --- | --- | --- |
| ***Second generation pills*** | |  |  |
|  | Ethinyl estradiol coupled with levonorgestrel (1) | 0.02/1 | Levesia (21 + 7) |
| ***Third generation pills*** | |  |  |
|  | Ethinyl estradiol coupled with gestodene (3) | 0.02/0.075 | Gestinyl (21 + 7) |
|  | Ethinyl estradiol coupled with gestodene (2) | 0.03/0.075 | Gestinyl (21 + 7) |
|  | Ethinyl estradiol coupled with desogestrel (2) | 0.02/0.15 | Mercilon and Daisynelle (21 + 7) |
| ***Fourth generation pills*** | |  |  |
|  | Ethinyl estradiol coupled with drospirenone (8) | 0.02/3 | Stefaminelle, Dizminelle, Yaz (24 + 4), Yasminelle, and Tasminetta (21 + 7) |
|  | Ethinyl estradiol coupled with drospirenone (5) | 0.03/3 | Yasmin, and Tasminetta (21 + 7) |
|  | Ethinyl estradiol coupled with dienogest (5) | 0.03/2 | Dienorette (21 + 7) |
|  | Estradiol (as hemihydrate) coupled with nomegestrol acetate (1) | 1.5/2.5 | Zoely |
| ***Other pills*** | |  |  |
|  | Ethinyl estradiol coupled with cyproterone acetate (2) | 0.035/2 | Vreya (21 + 7) |

**Supplementary Table S2 Hormone values exceeding both 1.5 × interquartile range and an absolute Z-score > 3 in eumenorrheic females (EUM) and in females using combined oral contraceptives (COC)**

| Participant | Total testosterone (nmol·L^–1^) | Free testosterone (pmol·L^–1^) | DHT  (nmol·L^–1^) | Androstenedione (nmol·L^–1^) | DHEA  (nmol·L^–1^) | DHEA-S (µmol·L^–1^) | SHBG  (nmol·L^–1^) |
| --- | --- | --- | --- | --- | --- | --- | --- |
| 1 (EUM) | 3.54 |  |  |  |  |  |  |
| 2 (EUM) |  | 37.03 | 7.22, 7.08 |  | 533.40, 532.96 |  |  |
| 3 (EUM) |  | 50.83 | 7.85 |  |  |  |  |
| 4 (EUM) |  |  |  |  |  | 12.60 |  |
| 5 (COC) |  | 34.11, 30.21 |  | 24.90 | 340.32 |  |  |
| 6 (COC) |  |  |  |  |  |  | 382 |
| 7 (COC) |  |  |  |  |  |  | 372 |
| 8 (COC) |  |  |  |  |  |  | 376 |

*DHT* dihydrotestosterone, *DHEA* dehydroepiandrosterone, *DHEA-S* dehydroepiandrosterone sulfate, *SHBG* sex hormone binding globulin

**Supplementary Table S3 Associations of androgen and SHBG concentrations with fat-free mass adjusted V̇O_2peak_ and F_max_ and with fat-free mass and fat mass adjusted CMJ**

|  | **Counter movement jump height (CMJ)** | | **Maximal bilateral isometric force production (F_max_)** | | **Aerobic capacity (V̇O_2peak_)** | |
| --- | --- | --- | --- | --- | --- | --- |
|  | **Std. estimate (95% CI)** | ***p*** | **Std. estimate (95% CI)** | ***p*** | **Std. estimate (95% CI)** | ***p*** |
| **Total testosterone** | 0.042 (-0.061, 0.145) | 0.424 | -0.024 (-0.121, 0.073) | 0.625 | 0.036 (-0.070, 0.141) | 0.506 |
| **Free testosterone** | -0.028 (-0.157, 0.101) | 0.670 | 0.001 (-0.119, 0.120) | 0.990 | -0.001 (-0.120, 0.119) | 0.989 |
| **Dihydrotestosterone** | -0.017 (-0.188, 0.153) | 0.841 | 0.040 (-0.117, 0.197) | 0.613 | -0.041 (-0.163, 0.082) | 0.511 |
| **Androstenedione** | 0.020 (-0.105, 0.144) | 0.756 | 0.002 (-0.101, 0.106) | 0.962 | -0.017 (-0.116, 0.081) | 0.730 |
| **DHEA** | 0.090 (-0.054, 0.235) | 0.220 | 0.065 (-0.070, 0.199) | 0.345 | -0.110 (-0.245, 0.025) | 0.110 |
| **DHEA-S** | 0.077 (-0.049, 0.204) | 0.230 | -0.010 (-0.129, 0.109) | 0.866 | -0.078 (-0.200, 0.044) | 0.208 |
| **SHBG** | 0.000 (-0.182, 0.181) | 0.999 | -0.128 (-0.299, 0.043) | 0.140 | 0.048 (-0.137, 0.232) | 0.610 |

*DHEA* dehydroepiandrosterone, *DHEA-S* dehydroepiandrosterone sulfate, *SHBG* sex hormone binding globulin

Estimates, confidence intervals (CI), and *p* values are from linear mixed-effects models with random intercepts for participants

**Supplementary Table S4 Differences in hormone associations with fat-free mass adjusted V̇O_2peak_ and F_max_ and with fat-free mass and fat mass adjusted CMJ in combined oral contraceptive using females compared with eumenorrheic females**

|  | **Counter movement jump height (CMJ)** | | **Maximal bilateral isometric force production (F_max_)** | | **Aerobic capacity (V̇O_2peak_)** | |
| --- | --- | --- | --- | --- | --- | --- |
|  | **Std. estimate (95% CI)** | ***p*** | **Std. estimate (95% CI)** | ***p*** | **Std. estimate (95% CI)** | ***p*** |
| **Total testosterone** | -0.001 (-0.215, 0.214) | 0.994 | -0.031 (-0.232, 0.170) | 0.760 | -0.156 (-0.373, 0.060) | 0.155 |
| **Free testosterone** | 0.240 (-0.089, 0.570) | 0.151 | -0.043 (-0.349, 0.263) | 0.782 | 0.046 (-0.259, 0.351) | 0.766 |
| **Dihydrotestosterone** | **0.450 (0.044, 0.855)** | **0.030** | 0.116 (-0.259, 0.491) | 0.540 | 0.261 (-0.031, 0.554) | 0.079 |
| **Androstenedione** | 0.060 (-0.207, 0.328) | 0.656 | 0.135 (-0.090, 0.360) | 0.235 | 0.000 (-0.209, 0.209) | 0.998 |
| **DHEA** | 0.046 (-0.250, 0.342) | 0.758 | 0.080 (-0.195, 0.355) | 0.568 | 0.079 (-0.199, 0.357) | 0.576 |
| **DHEA-S** | -0.001 (-0.301, 0.300) | 0.995 | 0.268 (-0.001, 0.537) | 0.051 | 0.029 (-0.249, 0.306) | 0.837 |
| **SHBG** | **0.721 (0.030, 1.411)** | **0.041** | -0.040 (-0.686, 0.606) | 0.903 | 0.200 (-0.453, 0.854) | 0.545 |

*DHEA* dehydroepiandrosterone, *DHEA-S* dehydroepiandrosterone sulfate, *SHBG* sex hormone binding globulin

Estimates (hormone and combined oral contraceptive use interactions), confidence intervals (CI), and *p* values are from linear mixed-effects models with random intercepts for participants

Significant associations are in bold
